# Threat Perception and Adaptive Capacity of Natural World Heritage Site Management

**DOI:** 10.1007/s00267-022-01780-y

**Published:** 2023-01-13

**Authors:** Martin Thomas Falk, Eva Hagsten

**Affiliations:** School of Business, Department of Business and IT, University of South-Eastern Norway, Campus Bø, Gullbringvegen 36, 3800 Bø, Norway

**Keywords:** Natural World Heritage sites, Threats, Adaptive capacity, Climate change, Ordered Probit model

## Abstract

This study offers new insights into the largest threats to natural and mixed World Heritage sites in developed countries as considered by their management. In addition to this, the capacity of the management to deal with threats is examined. An Ordered Probit model is used that distinguishes three groups of threats and four categories of adaptive capacity of the management. Data originate from the 2014 UNESCO Periodic Report II for sites in economically advanced countries (Europe, North America, Australia, New Zealand, Japan and South Korea) linked to the World Heritage Site database. Estimation results reveal that the probability of a major threat to World Heritage sites is perceived to be highest in the category of climate change and extreme weather events, followed by local conditions affecting the physical structure (temperature, rain, dust). Sites in tropical climates are perceived as significantly more threatened, as are those earlier listed as in danger. The likelihood of perceiving a major threat is highest in Turkey, Italy, Norway and North America. Threats related to climate change are those the management has the lowest capacity to deal with when other important aspects are controlled for. Large and natural areas have a higher perceived administrative capacity to deal with threats than others.

## Introduction

A changing climate as well as anthropogenic pressure poses increasing challenges to protected areas and national parks (Wang et al. 2015; Allan et al. 2017; Sabour et al. 2020). UNESCO Natural World Heritage sites are no exception and suffer from increasing forest fires and drought (Yellowstone National Park, Yosemite National Park, see Table 2), tree loss as well as a rising human pressure outside the protected area (Allan et al. 2017). Some high elevation Natural Heritage Sites are also affected by severe glacier retreats (for the Swiss Alps Jungfrau-Aletsch, see Bosson et al. 2019; Jasper National Park in the Canadian Rockies see Weber et al. 2019). Other Natural World Heritage Sites such as the Dolomites are losing their attractiveness for winter tourists due to the reduction of snow cover (Bonzanigo et al. 2016). Marine World Heritage Sites are threatened by the rise of the sea level, erosion, shoreline reduction and flooding (Dorset and East Devon Coast, Aeolian Islands, Wadden Sea) (Sabour et al. 2020). Additional aspects often highlighted include transportation infrastructure (Reddiar and Osti 2022 for natural WHS in Asia), pollution, physical resource extraction and social/cultural use of heritage sites (visitor pressure) as well as management and institutional aspects (Wang et al. 2015 for those in North America and Europe). Phillips (2015) points out that research is lacking on the understanding of to what extent current management practices can offset the impacts of climate change.

The aim of this study is twofold: (i) to examine threats to the UNESCO Natural and Mixed World Heritage Sites (hereafter labelled Natural World Heritage Sites) in economically advanced countries as perceived by their management and (ii) to explore the adaptive capacity of the management to deal with these threats. A wide range of potential threats as listed by the UNESCO (2014) in its periodic report is the basis for the analysis. These threats include buildings, ground transportation infrastructure, utilities, visitor impacts, environmental factors (pollution, waste and land conversion), climate change and sudden hazards. For the purpose of the analysis, information on 1145 threats to sites in the economically advanced countries (Europe, North America, Australia, New Zealand, Japan and South Korea) is linked to the UNESCO World Heritage database encompassing 83 natural and mixed sites. An Ordered Probit model is employed to estimate the relationship with specific threats as perceived by the managers as well as their capacity to deal with them. Explanatory variables include the year of inscription, size, selection criteria, inclusion on the danger list, kind of threat, climate zone and the country in which the site is located.

This study contributes to the growing literature on threats to natural and mixed World Heritage Sites based on information from UNESCO (Wang et al. 2015, Perry 2011; Valagussa et al. 2020, 2021; Birendra 2021). Unlike previous analyses that focus either on individual factors (Perry 2011; Valagussa et al. 2020) or a subset of endangered World Heritage Sites (Birendra 2021), this analysis uses a comprehensive dataset of Natural and Mixed World Heritage Sites for economically advanced countries together with a large number of presumptive threats. This allows more general conclusions to be drawn than from earlier studies. Another novelty is that both the aspects of importance for the intensity of threats and the adaptive capacity of the management to deal with them are modelled. The probability of a perceived threat is distinguished by three levels of intensity: negligible, minor and major while the adaptive capacity of the management is grouped into four categories (none, low, medium or high). Existing literature seldom considers site-specific characteristics in relation to threat intensity and the adaptive capacity of the management.

The structure of the study is as follows: the section ‘Conceptual background and previous literature’ outlines the conceptual background while the section ‘Empirical model’ describes the empirical approach. Data sources and descriptive statistics are presented in the section ‘Data and descriptive statistics’. The results are reported and discussed in the section ‘Empirical results’ and the section ‘Conclusions’ concludes.

## Conceptual Background and Previous Literature

### Threats to Natural World Heritage Sites

Many studies investigate different types of threats to Natural World Heritage Sites, commonly focussing on environmental or human-related aspects (Wang et al. 2015; Allan et al. 2017; Valagussa et al. 2020; see Table 1 for an overview). There are also analyses of the intensity of threats to Natural World Heritage Sites in North America and Europe based on the UNESCO period report II (Wang et al. 2015). In this case, the threats are reported for 13 categories including those internal as well as external to the sites. Results indicate that the intensity of threats is lowest for climate change and highest for management and institutional factors.

**Table 1:** Overview of threats to Natural World Heritage Sites

Authors	Name of site	Country	Kind of threat
*North America*			
Groulx et al. (2017)	Canadian Rocky Mountain Parks	Canada	Glacier retreat
Scott et al. (2007)	Canadian Rocky Mountain Parks	Canada	Climate change
			Forest fires
			Increase in visitation
Lemieux et al. (2018)	Canadian Rocky Mountain Parks	Canada	Glacier retreat
Allan et al. (2017)	Canadian Rocky Mountain Parks	Canada	Tree loss
Allan et al. (2017)	Wood Buffalo National Park	Canada	Tree loss
Turner et al. (1994, 1997)	Yellowstone National Park	United States	Wildfires
Meyer et al. (1992)	Yellowstone National Park	United States	Wildfires
			Drought
			De-vegetation by fire
Balling et al. (1992)	Yellowstone National Park	United States	Wildfires
Ireland et al. (2018)	Yellowstone National Park	United States	Drought
			Increased temperature
			Reduced snowpack
			Longer growth season
			Wildfire
Shafer (2012)	Yellowstone National Park	United States	Geothermal drilling, oil and gas leasing close by
			Residential development outside
Allan et al. (2017)	Yellowstone National Park	United States	Tree loss
Pearlstine et al. (2010)	Everglades National Park	United States	Sea level rise
			Increased temperature
			more extreme storm events
			Extended droughts
			Increased atmospheric CO_2_ concentration
Kushlan (1987)	Everglades National Park	United States	Water regulation system
Choe and Schuett (2020)	Everglades National Park	United States	Overpopulation
			Urban development
			Environmental damage
			Water inflow
			Natural disasters
Perry (2011)	Everglades National Park	United States	Sea level rise
Leung and Marion (1999)	Great Smoky Mountains National Park	United States	Recreation and tourism visitation
Bradley et al. (2021)	Great Smoky Mountains National Park	United States	Pesticides and pharmaceuticals in protected streams
Moritz et al. (2008)	Yosemite National Park	United States	High-altitude species are under threat,
Lutz et al. (2010)	Yosemite National Park	United States	Declining water availability has a negative impact on tree species
Scholl and Taylor (2010)	Yosemite National Park	United States	Wildfire
White (2007)	Yosemite National Park	United States	Traffic congestion
Guarín and Taylor (2005)	Yosemite National Park	United States	Tree mortality due to drought
Allan et al. (2017)	Grand Canyon National Park	United States	Tree loss
Cole (1990)	Grand Canyon National Park	United States	Destruction of the soil crust
Fulé and Laughlin (2007)	Grand Canyon National Park	United States	Wildfires
Shafer (2012)	Grand Canyon National Park	United States	Water utilities
Selkoe et al. (2009)	Papahānaumokuākea	United States	Alien species
			Bottom fishing
			Increased UV radiation
			Lobster fishing
			Marine debris
			Sea level rise
			Seawater acidification
			Ship-based pollution
*Europe*			
Mandić (2021)	Plitvice Lakes National Park	Croatia	Visitation pressure
			Mass tourism
Allan et al. (2017)	Mount Athos	Greece	Tree loss
Perry (2011)	Isole Eolie	Italy	Sea level rise
			Intensive infrastructure
Lo Piccolo et al. (2012)	Isole Eolie	Italy	Mass tourism
Maramai et al. (2005)	Isole Eolie	Italy	Tsunamis
Selva et al. (2020)	Isole Eolie	Italy	Volcanoes
Bonzanigo et al. (2016)	The Dolomites	Italy	Water consumption for snowmaking
			Decline in snow cover
			Climate change
Balbi et al. (2013)	The Dolomites	Italy	Decline in snow cover
			Climate change
Franch et al. (2005)	The Dolomites	Italy	Pollution
			Mass tourism
Scuttari et al. (2019)	The Dolomites	Italy	Mass tourism
			Visitation pressure
Oklevik et al. (2019)	West Norwegian Fjords – Geirangerfjord and Nærøyfjord	Norway	Visitation pressure
			Mass tourism
Armaş and Avram (2009)	Danube Delta	Romania	Floods
Sabour et al. (2020)	Danube Delta	Romania	Damming of river sediments upstream of the delta
Hampton et al. (2008)	Lake Baikal	Russia	Climate change
			Increase in temperatures
Allan et al. (2017)	Lake Baikal	Russia	Tree loss
Dickson et al. (1987)	Teide National Park	Spain	Invading plants
González et al. (2019)	Teide National Park	Spain	Mass tourism
Olano et al. (2017)	Teide National Park	Spain	Extreme drought events
Perry (2011)	Teide National Park	Spain	Intense infrastructure
Allan et al. (2017)	Doñana National Park	Spain	Tree loss
Palomo et al. (2014)	Doñana National Park	Spain	land use changes occurred outside it
			Increase in irrigated agricultural lands
			Increase in urbanised areas
			Decrease in wetlands surface
Serrano and Serrano (1996)	Doñana National Park	Spain	Water utilities
Fernández-Ayuso et al. (2018)	Doñana National Park	Spain	Decreasing trend of groundwater
Bianchi (2002)	Garajonay National Park	Spain	Mass tourism
			Visitation pressure
Bosson et al. (2019)	Alps Jungfrau‐Aletsch	Switzerland	Glacier retreat
Salim et al. (2022)	Alps Jungfrau‐Aletsch	Switzerland	Glacier retreat
Dilsiz (2002)		Turkey	Mass tourism
			Degradation
			Pollution
Allan et al. (2017)	St. Kilda	United Kingdom	Human pressure
Cigna et al. (2018)	Giant’s Causeway and Causeway Coast	United Kingdom	Landslide hazard
*Australia, Japan, New Zealand and South Korea*			
Kroon et al. (2016)	Great Barrier Reef	Australia	Land-based pollution
			Decline in reef water quality
Lewis et al. (2009)	Great Barrier Reef	Australia	Pesticides (insecticides, herbicides and fungicides)
Hughes et al. (2015)			Unsustainable fishing
			Runoff from agriculture
			Development of coastal areas
			Rapid climate change
Ainsworth et al. (2007)	Great Barrier Reef	Australia	Coral bleaching disease
Wright et al. (2011)	Greater Blue Mountains Area	Australia	Water pollution
Chapple et al. (2011)	Greater Blue Mountains Area	Australia	Wild dogs and fire
Hadwen et al. (2003)	Fraser Island	Australia	Tourism
Burns and Howard (2003)	Fraser Island	Australia	Tourism
Dixon et al. (2004)	Tasmanian Wilderness	Australia	Deterioration of walking tracks
Bradshaw et al. (2007)	Kakadu National Park	Australia	Non-indigenous animal species
Turton (2005)	Wet Tropics of Queensland	Australia	Recreation and tourism
Priddel et al. (2006)	Lord Howe Island	Australia	Increased urbanisation
Sabour et al. (2020)	Te Wahipounamu	New Zealand	Sea level rise
			Shoreline change
Nguyen et al. (2022)	Ogasawara Islands	Japan	Tourism development
Shoyama and Braimoh (2011)	Shiretoko	Japan	Reforestation
*Transboundary*			
Serrano and Serrano (1996)	Wadden Sea	Denmark, Germany, Netherlands	Accelerated sea level rise
			Increasing air and water temperatures
			Storm
Thieltges et al. (2013)	Wadden Sea	Denmark, Germany, Netherlands	Species invasions, climate change
Sabour et al. (2020)	Wadden Sea	Denmark, Germany, Netherlands	Reduction of the coastline through sand flushing
Munaretto and Klostermann (2011)	Wadden Sea	Denmark, Germany, Netherlands	Sea level rise
			Increased temperature of seawater
			Loss of attractiveness for many species
Kabat et al. (2012)	Wadden Sea	Denmark, Germany, Netherlands	Sea level rise
Hofstede (2019)	Wadden Sea	Denmark, Germany, Netherlands	Sea level rise
			Floods
Perry (2011)	High Coast/Kvarken Archipelago	Sweden/Finland	Sea level rise
Allan et al. (2017)	Waterton Glacier International Peace Park	Canada/United States	Tree loss
Żmihorski et al. (2018)	Białowieża Forest	Transboundary	Bark beetle outbreak
Wang et al. (2015)	All natural WHS as of 2004	All NWHS countries	13 factors from the UNESCO period report
Birendra (2021)	WHS in danger		Factors from the UNESCO period report
Valagussa et al. (2020)	West Norwegian Fjords – Geirangerfjord and Nærøyfjord	Norway	Earthquake
	Pirin National Park	Bulgaria	Volcanic eruption
	Swiss Tectonic Arena Sardona	Switzerland	Avalanche/landslide
	Caves of Aggtelek Karst and Slovak Karst	Hungary, Slovakia	Flooding
	Gulf of Porto: Calanche of Piana, Gulf of Girolata, Scandola Reserve	France	Tsunami/tidal wave
	Donana National Park	Spain	Storm
	Pitons, cirques and remparts of	Reunion France	Change to oceanic water
	Srebarna Nature Reserve	Bulgaria	Fire (wild)
	Mount Etna	Italy	Desertification
	Isole Eolie (Aeolian Islands)	Italy	Erosion and siltation/deposition
			Drought
			Temperature change
			Other climate change impacts

Source: see reference list

Tree loss, climate change, and forest fires endanger Natural World Heritage Sites in mountainous areas such as the Yellowstone Natural Park, the Waterton Glacier International Peace Park and Greater Blue Mountains Area (Chapple et al. 2011; Allan et al. 2017; Rammer et al. 2021). Studies for other natural parks demonstrate forest losses in a range of 5–12% over the period 2000–2012 (Wood Buffalo National Park, Grand Canyon National Park, Doñana National Park, Yellowstone National Park, Mount Athos and Canadian Rocky Mountain Parks) (Allan et al. 2017).

Glacier retreat poses a serious threat to sites at higher elevations in North America (Groulx et al. 2017; Lemieux et al. 2018; Weber et al. 2019) as well as in Europe (Bosson et al. 2019; Salim et al. 2022) and a rise of the sea level is hazardous for sites along the coast (Sabour et al. 2020). Alien species are less commonly reported as being harmful to national parks. An exception to this is the outbreak of the bark beetle (Żmihorski et al. 2018) and the increase in non-indigenous animal species in the Kakadu National Park in Australia (Bradshaw et al. 2007). The threat caused by water utilities is also frequently listed (Serrano and Serrano 1996 for instance). Land-based pollution, runoff from agriculture (pesticides in form of insecticides, herbicides and fungicides) leading to a decline in reef water quality is seen as a major threat to the Great Barrier Reef in Australia (Lewis et al. 2009; Kroon et al. 2016).

Natural World Heritage Sites are commonly exposed to high numbers of visitors (Buckley 2018) or are located close to mass tourism destinations (Teide National Park, Doñana National Park, Isole Eolie and the Dolomites). Many popular Natural World Heritage sites in North America and in Europe are subjected to mass tourism (Dolomites and Plitvice Lakes National Park, Yosemite National Park) (Mandić 2021; Scuttari et al. 2019; White 2007). Mandić (2021), for instance, documents that the amount of visitors in Plitvice Lakes National Park disables the internal infrastructure and Adie (2019) demonstrates that the Yellowstone National Park suffers from large crowds of people on the walkways. Similar evidence is reported for the Australian national parks (Fraser Island, Tasmanian Wilderness, Wet Tropics of Queensland, see Hadwen et al. 2003; Burns and Howard 2003; Dixon et al. 2004; Turton 2005) and the Ogasawara Islands in Japan (Nguyen et al. 2022).

Previous research shows that the inscription to the UNESCO Natural World Heritage Sites list may lead to an increased flow of tourists (Buckley 2018; Yang et al. 2019). Recent literature emphasises the risk of over-tourism even for relatively remote Natural World Heritage Sites such as fjords in Norway, especially in relation to cruising ships (Oklevik et al. 2019). The latest IUCN Guidelines for Tourism and Visitor Management in Protected Areas agree that excessive tourism has negative ecological, socio-cultural and economic impacts (Leung et al. 2018).

There are also other human pressure-related factors that are listed as threats. These include air pollution, waste, land conversion, traffic and residential development (Levin et al. 2019; Birendra 2021). In the example of the St Kilda Natural World Heritage Site in Scotland, the human footprints are considered to include the built environment, arable land, grazing land, population density, night-time lighting, railways, major roads and navigable waterways (Allan et al. 2017).

Thus, several studies assess the threats to natural World Heritage sites in terms of hazards (man-made or not), climate change and sudden geological events. However, most studies only look at a smaller group of threats, are often limited to specific sites and do not consider area-specific characteristics, despite the fact that the latter may vary vastly across sites. Whether the Natural World Heritage Site in question is classified as in danger is less often contemplated together with other aspects (Birendra 2021).

Typically, the size of the Natural World Heritage site may be another important feature. Large, protected areas may be more resilient and able to withstand gradual or sporadic major changes such as fires and geological hazards (Hockings 2006). Natural World Heritage sites that were inscribed early are long since commercialised, presumably better known to potential visitors and because of this more likely to be at risk from several perspectives (Falk and Hagsten 2022). This implies that the age of the UNESCO label is important.

Many Natural World Heritage Sites are located in areas where sudden geological events are more likely to occur (Valagussa et al. 2020), something that makes them more vulnerable. The severity of the threat itself is also pertinent (Wang et al. 2015). Sudden events such as earthquakes may occur infrequently, but when they do, they are more devastating. Climate zone is another possible deciding factor behind presumptive threats to the Natural World Heritages. Sites in the polar region are less accessible and possibly suffer fewer direct human impacts, while at the same time, climate change is accelerating in this particular region (Constable et al. 2022). Natural World Heritage Sites in tropical areas, on the other hand, are at risk from human-induced threats such as deforestation and transportation infrastructure (Allan et al. 2017).

Based on the conceptual considerations, four hypotheses are formulated on aspects of importance for how the management perceives the threats to the Natural World Heritage Site:

H1: The likelihood of a major threat to the Natural World Heritage Site is related to its location.

H2: The likelihood of a major threat to the Natural World Heritage Site is related to its characteristics.

H3: The likelihood of a major threat to the Natural World Heritage Site is related to the kind of threat.

H4: The likelihood of a major threat is higher for factors external to the Natural World Heritage Site.

### Capacity of the Management to Respond to the Threats

A related research question to the severity of threats to the Natural World Heritage Site is the capacity of its management to respond to them. Adaptive capacity can be defined in different ways, but the climate change literature, for instance, describes it as the potential or ability of a system to adapt to threats such as climate change (Smit and Wandel 2006). According to Engle (2011), institutions, governance and management play a major role in this ability. Nelson et al. (2007) suggest that adaptive capacity is a prerequisite for a system to handle disruptions like environmental or climate change. The adaptive capacity of institutions can be considered a function of local management practices (including scientific and technical support) capital, knowledge, technology, infrastructure as well as social or intellectual capital and the effectiveness of these practices in managing the impacts of change (Nelson et al. 2007). Chapple et al. (2011) distinguish between conventional and adaptive management strategies and apply this to overcome persistent challenges in the Greater Blue Mountains.

Several studies examine the capacity of site managers and stakeholders to adapt to climate change adaptation or other related threats and the perceived ability to implement and operationalise such strategies. For instance, Lemieux and Scott (2011) analyse climate change adaptation options for protected area authorities in Canada based on a panel of experts. Institutional capacity to implement and manage the challenges related to climate change is distinguished into four classes (i) capacity to implement and manage definitely exists, (ii) capacity to implement and manage exists or could be readily enhanced, (iii) capacity to implement and manage does not exist and difficult to enhance (v) capacity to implement and manage definitely does not exist. The results show a high level of agreement on the desirability of adaptation options, although the implementation capacity is perceived to be low. The Canadian agencies responsible for planning and managing protected areas are also found to have limited capacity to implement decisions related to climate change (Lemieux et al. 2011). Considering Natural World Heritage Sites along the coast, climate-related threats do not offer easily manageable solutions at the regional level since the problem is global (Selkoe et al. 2009). An examination of 229 management plans for Natural and Mixed World Heritage Sites reveals that only 42% of them have such a plan and that 28% have comprehensive up-to-date tourism planning (Job et al. 2017).

As highlighted in literature, several factors challenge the efficient and effective integration of climate change adaptation into decision-making and management: institutional and policy-oriented (lack of a clear mandate to adapt to climate change), financial and human (insufficient human and financial resources), informational (insufficient research and monitoring programmes) and contextual (sufficient regional networks to mitigate potential transboundary impacts) (Lemieux et al. 2013).

Based on the literature on management capacity to deal with threats and by considering the same independent variables of importance as for the threats, the fifth hypothesis is formulated:

H5: The management adaptive capacity is lower for threats external to the Natural World Heritage Site.

## Empirical Model

The threats to Natural World Heritage Sites at the individual level are modelled as a function of site characteristics such as size and age, listed as in danger, criteria for inclusion, climate zone, country dummy variables and dummy variables for category of threat. This latter variable makes it possible to investigate whether the intensity differs significantly across factors. The dependent variable of the model is the perception of threats to the Natural (and Mixed) World Heritage Site as reported to UNESCO by their management or responsible institutions. This perception is measured on an ordinal scale of threats: (1) major, (2), minor and (3) negligible. In order to account for the nature of the dependent variable the Ordered Probit model is employed (Greene 2017). This means that the latent variable, $Y_{1ij}^ \ast$, measuring the probability of the intensity of threat is specified as follows:

1 $$\begin{array}{l}Y_{1ij}^ \ast = \beta _{10} + \beta _{11}{{{\mathrm{ln}}}}\left( {Size} \right)_j +\, \mathop {\sum }\limits_{a = 1}^3 \beta _{12a}Age_j^a + \beta _{13}Dangerlist_j \\ \qquad \quad+\, \beta _{14}Mixed_j + \mathop {\sum }\limits_{k = 1}^{11} \beta _{15k}Kind_{ij} +\mathop {\sum }\limits_{z = 1}^4 \beta _{16z}Climatezone_{jz} \\\qquad \quad+ \mathop {\sum }\limits_{s = 1}^4 \beta _{17s}Criteria_{is}+ \mathop {\sum }\limits_{c = 1}^{20} \beta _{18c}Country_{cj} + \varepsilon _{1ij},\end{array}$$

$$Y_{1ij} = \left\{ \begin{array}{l}0\,{{{\mathrm{if}}}} - \infty \,<\, Y_{1ij}^ \ast \le \gamma _1\left( {{{{\mathrm{negligible}}}}} \right)\\ 1\,{{{\mathrm{if}}}}\,\gamma _{{{\mathrm{1}}}}\, < \,Y_{1ij}^ \ast \le \gamma _2\left( {{{{\mathrm{minor}}}}} \right)\\ 2\,{{{\mathrm{if}}}}\,\gamma _{{{\mathrm{2}}}}\, < \,Y_{1ij}^ \ast < + \infty \left( {{{{\mathrm{major}}}}} \right)\end{array} \right\}$$

where *γ*_1_, *γ*_2_, are the cutoff points of the distribution of the latent measure of threat perception, which is to be estimated along with *β*, the vector of coefficients. The subscript *i* (1,..,1145) is the perception of threat while *j* (1,..,83) represents the site in question. Variable *c* is the country in which the site is located including transboundary areas, *ε*_1*ij*_ reflects the error term and *β*_0_ is the constant. The latent response variable representing the severity of the threat is labelled $Y_{1ij}^ \ast$, ln() is the natural logarithm and *Size* denotes the area of the site in hectares excluding the wider zone. *Age* is measured as a set of dummy variables for the inscription year until 2014 and *Dangerlist* is a dummy variable equal to one if the site is listed as being in danger. An additional dummy variable *Mixed* separates out sites that are also cultural. *Kind* is a set of dummy variables representing 11 categories of threats as defined in the UNESCO period report II with pollution as the reference group (see also Wang et al., 2015). Variable *Climatezone* constitutes a set of dummy variables for tropical (A), dry (B), continental (D) and polar (E) areas, with temperate (C) as the reference category and *Criteria* is a set of dummy variables for the inscription. A set of *Country* dummy variables controls for country-specific factors including transboundary.

In the second stage of the empirical analysis, aspects of importance for the adaptive capacity of the management are dealt with. This dependent variable corresponds to the definition used by Lemieux and Scott (2011) and consists of the four ordered categories no, low, medium or high management capacity to deal with the threats. By employing the same independent variables as in Eq. (1), the probability of the adaptive capacity of the management to deal with the threat $Y_{2ij}^ \ast$, can be written as follows:

2 $$\begin{array}{l}Y_{2ij}^ \ast = \beta _{20} + \beta _{21}{{{\mathrm{ln}}}}\left( {Size} \right)_j + \mathop {\sum }\limits_{a = 1}^3 \beta _{22a}Age_j^a + \beta _{23}Dangerlist_j \\ \qquad\quad +\, \beta _{24}Mixed_j + \mathop {\sum }\limits_{k = 1}^{11} \beta _{25k}Kind_{ij} + \mathop {\sum }\limits_{z = 1}^4 \beta _{26z}Climatezone_{jz} \\ \qquad\quad+ \mathop {\sum }\limits_{s = 1}^4 \beta _{27s}Criteria_{is} + \mathop {\sum }\limits_{c = 1}^{20} \beta _{28c}Country_{cj} + \varepsilon _{2ij},\end{array}$$

$$Y_{2ij} = \left\{ {\begin{array}{ll} {\begin{array}{*{20}{l}} 0 & {{{{\mathrm{if}}}}\, - \infty \,<\, Y_{2ij}^ \ast \le \gamma _1\left( {{{{\mathrm{no}}}}\,{{{\mathrm{capacity}}}}} \right)} \end{array}} \\ {\begin{array}{*{20}{l}} 1 & {{{{\mathrm{if}}}}\,\gamma _1 \,<\, Y_{2ij}^ \ast \le \gamma _2\left( {{{{\mathrm{low}}}}\,{{{\mathrm{capacity}}}}} \right)} \end{array}} \\ {\begin{array}{*{20}{l}} 2 & {{{{\mathrm{if}}}}\,\gamma _2 \,<\, Y_{2ij}^ \ast \le \gamma _3\left( {{{{\mathrm{medium}}}}\,{{{\mathrm{capacity}}}}} \right)} \end{array}} \\ {\begin{array}{*{20}{l}} 3 & {{{{\mathrm{if}}}}\,\gamma _3\, <\, Y_{2ij}^ \ast \,<\, + \infty \left( {{{{\mathrm{high}}}}\,{{{\mathrm{capacity}}}}} \right)} \end{array}} \end{array}} \right\}$$

where *γ*_1_, *γ*_2_ and *γ*_3_ are the cutoff points of the distribution of the latent measure of adaptive capacity. The ordered response model is estimated using maximum likelihood techniques (Greene 2017). Cluster-adjusted standard errors at the World Heritage Site level are used to account for the possibility that individual threats to a separate site may not be independent of each other. Not only the directions of the presumptive relationships, but also the marginal effects for the two equations and each of the categories will be estimated.

## Data and Descriptive Statistics

The estimations make use of information from the UNESCO World Heritage Site database (http://whc.unesco.org/en/list) linked to the 2014 UNESCO Periodic Report II. For the 2014 reporting round, information is available for sites in North America, Europe, Asia and Oceania. In order to obtain a homogeneous database, the study focuses on sites located in economically advanced countries (Europe and OECD countries in North America, Oceania and Asia).

The database used for the analysis includes 1145 threats to 83 Natural and Mixed World Heritage sites in Europe, North America, Australia, New Zealand, Japan and South Korea (including the Asian part of Russia and Turkey as well as overseas sites belonging to France and the United Kingdom). Originally, there are 93 sites in the database, but ten of those do not report threats and hence are excluded from the analyses: Isole Eolie (Aeolian Islands), Mount Etna, Natural System of Wrangel Island Reserve, Lena Pillars Nature Park, Laponian Area, Redwood National and State Parks, Monte San Giorgio, Ogasawara Islands Bonin Islands, Shirakami-Sanchi and Ningaloo Coast). In three cases, there is a statement that ‘No factor is both current and negative’. Data on size (surface in hectares), year of inscription, country, kind of site and inclusion in the danger list originates from the UNESCO world heritage database. Information on selection criteria refers to UNESCO World Heritage Centre – The Criteria for Selection (http://whc.unesco.org/en/criteria/). Four criteria for natural sites are considered:

N7: to contain superlative natural phenomena or areas of exceptional natural beauty,

N8: to be outstanding examples representing major stages of earth’s history,

N9: to be outstanding examples representing significant on-going ecological and biological processes as well as

N10: to contain the most important and significant natural habitats for in-situ conservation of biological diversity.

Each World Heritage Site is requested to submit a periodic report on the state of conservation of its territory and properties (https://whc.unesco.org/en/periodicreporting/). Information on climate zone is linked to the dataset using information on latitudes and longitudes (World Maps of Köppen-Geiger climate classification http://koeppen-geiger.vu-wien.ac.at/present.htm). The Köppen-Geiger climate system encompasses 22 zones of which the five main categories are used (Tropical zone A to polar E).

The 2014 periodic reporting Cycle 2 (PR-II, 2008-2015) is covering a pre-determined six-to-ten-year cycle and includes a series of questions on the management organisation as well as on ‘factors and threats affecting the property’, and the intensity of threats. For instance, Wang et al. (2015) use 13 factors to address the intensity of threats. The information on threats is based on a self-assessment of the responsible institutions by means of a questionnaire including four categories: (1) catastrophic, (2) significant, (3) minor and (4) insignificant. Since the category ‘catastrophic’ rarely occurs, the first two categories are merged and relabelled as a ‘major’ threat, while the category insignificant is re-named ‘negligible’ to avoid confusion with econometric terminology.

A limitation is that questions related to threats from wars and conflicts are not included in the survey. This might be less relevant to the time period and area covered by the study (Europe, North America and other OECD countries). In developing countries, Levin et al. (2019) conclude that conflicts and wars are one of the main threats to Natural World Heritage Sites. Besides the intensity of the threat, there is also information on the spatial and temporal scale as well as the management capacity to deal with the threats distinguished by four categories (no, low, medium and high).

Descriptive statistics based on the 83 Natural World Heritage Sites show that the most common threats, Pollution, Physical resource extraction, Climate change and severe weather events as well as Invasive/alien species or hyper-abundant species, are all except pollution also dominant in the category ‘major threats’. Threats from Climate change and severe weather events, Invasive/alien species or hyper-abundant species or Local conditions affecting physical fabric (temperature, rain and dust) are perceived as major to 58, 49 and 43%, respectively (Table 2). Negligible and minor threats more often relate to issues closer to the administration of the site such as buildings and services. These statistics contrast with those reported by Wang et al. (2015) who use information from the 2004 wave of the UNESCO periodic report 2004.

**Table 2:** Descriptive statistics threats to Natural World Heritage Sites

	Intensity of threat			
	Negligible	Minor	Major	Number of threats
Buildings and development	28	65	8	65
Transportation infrastructure	30	53	16	105
Services infrastructures	34	50	16	105
Pollution	32	49	20	123
Biological resource use/modification	31	57	12	122
Physical resource extraction	24	57	19	37
Local conditions affecting physical fabric (temperature, rain, dust)	14	43	43	72
Social/cultural uses of heritage (tourism/visitor/recreation)	20	53	28	112
Illegal activities	29	44	27	70
Climate change and severe weather events	12	30	58	120
Sudden ecological or geological events	23	40	38	96
Invasive/alien species or hyper-abundant species	22	29	49	118
Total	25	46	29	1145

Relates to 83 UNESCO Natural World Heritage and Mixed Site database

Source: UNESCO Periodic report II and World heritage database

A reasonably high management capacity to adapt to threats can be observed for services Infrastructures, building and development, biological resource use/change and physical resource extraction, all approximately in one out of three cases (Table 3). Absolutely lowest capacity is found for Illegal activities and Physical resource extraction. Climate change is perceived to be possible to deal with for one out of ten sites. This coincides with findings by Lemieux et al. (2011).

**Table 3:** Descriptive statistics on adaptive capacity of management to deal with threats

	Capacity				
	None	Low	Medium	High	Number of threats
Buildings and development	3	15	56	26	65
Transportation infrastructure	2	21	41	36	105
Services infrastructures	11	26	48	15	105
Pollution	3	26	37	34	123
Biological resource use/modification	5	32	30	32	122
Physical resource extraction	25	33	28	14	37
Local conditions affecting physical fabric (temperature, rain, dust)	2	22	56	20	72
Social/cultural uses of heritage (tourism/visitor/recreation)	4	27	47	21	112
Illegal activities	34	38	23	5	70
Climate change and severe weather events	11	29	38	22	120
Sudden ecological or geological events	8	24	62	6	96
Invasive/alien species or hyper-abundant species	10	26	43	21	118
Total	3	15	56	26	1145

Relates to 83 UNESCO Natural World Heritage sites

Source: UNESCO Periodic report II and World Heritage Site database

The average size of a Natural World Heritage Site is 2.7 million hectares (equal to 27,000 square kilometres) (Table 4). Inclusion in the danger list is rare, only four out of the 83 sites in the dataset are closely acquainted with this list before 2014 (Everglades National Park, Plitvice Lakes National Park, Srebarna Nature Reserve and Yellowstone National Park). Every fourth Natural World Heritage Site is between one and 15 years old as inscribed (based on the reference year 2014).

**Table 4:** Descriptive statistics independent variables

	Mean/%			Mean/%
Size in hectares	2,702,383	Australia	au	23.0
1–15 years (as of 2014)	24.1	Bulgaria	bg	3.2
16–26 years	24.5	Canada	ca	2.0
27–32 years	26.1	Switzerland	ch	2.2
33–36 years	25.2	Denmark	dk	1.5
Danger list	8.9	Spain	es	3.5
Mixed site	19.6	France	fr	5.0
Köppen Climate Classification A	17.5	United Kingdom	uk	2.0
Köppen Climate Classification B	8.6	Greece	gr	0.8
Köppen Climate Classification C	45.9	Croatia	hr	2.2
Köppen Climate Classification D	19.6	Italy	it	0.6
Köppen Climate Classification E	8.6	Japan	jp	0.9
Criterion N7	72.1	Norway	no	2.4
Criterion N8	68.4	New Zealand	nz	5.6
Criterion N9	54.8	Portugal	pt	0.6
Criterion N10	56.6	Romania	ro	0.8
Buildings and development	5.7	Russia	ru	8.6
Transportation infrastructure	9.2	Slovenia	si	0.5
Services infrastructure	9.2	Turkey	tr	2.6
Pollution	10.7	USA	us	20.6
Biological resource use/modification	10.7	Transboundary		10.7
Physical resource extraction	3.2	Other (de, is, kr, me)		0.7
Local conditions affecting physical fabric (temperature, rain, dust)	6.3			
Social/cultural uses of heritage (tourism/visitor/recreation)	9.8			
Illegal activities	6.1			
Climate change and severe weather events	10.5			
Sudden ecological or geological events	8.4			
Invasive/alien species or hyper-abundant species	10.3			

Source: UNESCO Periodic report II and World Heritage Site database

## Empirical Results

The Ordered Probit estimations reveal that the intensity of threats depends significantly on the country, climate zone, selection criteria and category of threat. Climate change and severe weather events attain the highest probability for the perception of a major threat (Table 5, marginal effects and Table 7 in Appendix, for coefficients). A lack of ability to adapt to these same threats receives the highest marginal effects in the estimations of the management capacity (Table 6 and Table 8 in Appendix).

**Table 5:** Ordered Probit estimations of the perception of threats to Natural World Heritage Sites (marginal effects)

	Negligible		Major	
	dy/dx	z-stat	dy/dx	z-stat
Log size in hectares	−0.006	−0.68	0.006	0.68
0–15 years (16–26 reference category)	0.030	0.49	−0.031	−0.49
27–32 years	−0.003	−0.07	0.004	0.07
33–36 years	−0.007	−0.15	0.007	0.15
Danger list	−0.168***	−2.97	0.173***	3.01
Mixed WHS	0.051	1.58	−0.053	−1.58
Buildings and development (reference pollution)	0.007	0.16	−0.007	−0.16
Transportation infrastructure	−0.043	−0.94	0.044	0.93
Services infrastructure	−0.004	−0.10	0.004	0.10
Biological resource use/modification	−0.035	−0.78	0.036	0.78
Physical resource extraction	−0.025	−0.52	0.026	0.52
Local conditions affecting physical fabric (temperature, rain, dust)	−0.201***	−3.71	0.207***	3.74
Social/cultural uses of heritage (tourism/visitor/recreation)	−0.148***	−2.83	0.152***	2.85
Illegal activities	−0.091**	−2.01	0.094**	2.00
Climate change and severe weather events	−0.309***	−6.81	0.318***	7.03
Sudden ecological or geological events	−0.193***	−3.26	0.199***	3.31
Invasive/alien species or hyper-abundant species	−0.217***	−3.99	0.224***	4.11
Köppen Climate Classification A (ref. cat. ‘C’)	−0.151***	−3.58	0.155***	3.45
Köppen Climate Classification B	−0.043	−1.11	0.045	1.11
Köppen Climate Classification D	0.137**	2.18	−0.142**	−2.24
Köppen Climate Classification E	0.033	0.39	−0.034	−0.40
Criterion N7	0.068**	2.33	−0.070**	−2.43
Criterion N8	0.061	1.60	−0.063	−1.57
Criterion N9	0.080**	2.08	−0.082**	−2.09
Criterion N10	−0.027	−0.71	0.027	0.71
Australia, AU (reference all other countries)	−0.070	−0.74	0.072	0.73
Bulgaria, BG	0.225**	2.42	−0.232**	−2.46
Canada, CA	−0.279***	−3.09	0.287***	3.11
Switzerland, CH	−0.271**	−2.17	0.280**	2.18
Denmark, DK	−0.239**	−2.02	0.246**	2.06
Spain, ES	−0.156	−1.62	0.161	1.62
France, FR	0.087	0.92	−0.090	−0.92
United Kingdom, UK	−0.076	−0.85	0.078	0.85
Greece, GR	−0.005	−0.04	0.005	0.04
Croatia, HR	−0.272**	−2.22	0.281**	2.18
Italy, IT	−0.363***	−3.77	0.375***	3.91
Japan, JP	−0.178*	−1.83	0.183*	1.86
Norway, NO	−0.334***	−3.50	0.344***	3.63
New Zealand, NZ	0.078	0.66	−0.080	−0.67
Portugal, PT	−0.046	−0.53	0.048	0.53
Romania, RO	−0.024	−0.25	0.025	0.25
Russia, RU	−0.047	−0.37	0.049	0.37
Slovenia, SI	−0.189**	−2.14	0.195**	2.13
Turkey, TR	−0.471***	−3.65	0.486***	3.68
USA, US	−0.196**	−2.19	0.202**	2.15
Transboundary	−0.002	−0.02	0.002	0.02
Number of observations	1145			
Number of WHSs (natural and mixed)	83			

The average marginal error dy/dx is calculated for the three categories negligible, minor and major threat. An Ordered Probit model is estimated using the Stata command ‘oprobit’ and the model option ‘clust(id)’ where id is the world heritage sites. Coefficients and z-stat are displayed in Table 7 in Appendix. The marginal effects for the middle category ‘Minor’ are not displayed as no variables are significant

***, ** and * indicate statistical significance at the 1, 5 and 10% levels

**Table 6:** Ordered Probit estimations of the management capacity to deal with threats

	High		Medium		Low		None	
	dy/dx	z-stat	dy/dx	z-stat	dy/dx	z-stat	dy/dx	z-stat
Log size in hectares	0.03**	2.01	0.01	1.56	−0.02*	−1.85	−0.02**	−2.01
0–15 years (16–26 ref. category)	0.02	0.25	0.01	0.24	−0.01	−0.25	−0.01	−0.25
27–32 years	0.03	0.38	0.01	0.37	−0.02	−0.38	−0.02	−0.38
33–36 years	0.00	−0.01	0.00	−0.01	0.00	0.01	0.00	0.01
Danger list	0.13**	2.12	0.03*	1.90	−0.09**	−2.01	−0.07**	−2.27
Mixed WHS	−0.15***	−3.20	−0.04**	−2.32	0.10***	3.03	0.08***	3.14
Buildings and development (reference pollution)	0.00	0.07	0.00	0.07	0.00	−0.07	0.00	−0.07
Transportation infrastructure	−0.04	−1.21	−0.01	−1.31	0.03	1.22	0.02	1.26
Services infrastructure	−0.14***	−4.14	−0.04***	−2.91	0.10***	4.17	0.08***	3.94
Biological resource use/modification	−0.03	−0.88	−0.01	−0.89	0.02	0.87	0.02	0.90
Physical resource extraction	−0.06	−1.26	−0.02	−1.21	0.04	1.27	0.03	1.25
Local conditions affecting physical fabric (temperature, rain, dust)	−0.23***	−4.71	−0.06***	−2.85	0.16***	4.39	0.13***	4.46
Social/cultural uses of heritage (tourism/visitor/recreation)	−0.08*	−1.96	−0.02**	−2.00	0.05**	1.98	0.05**	2.03
Illegal activities	−0.09*	−1.89	−0.02*	−1.78	0.06*	1.87	0.05*	1.94
Climate change and severe weather events	−0.34***	−7.31	−0.09***	−3.30	0.23***	6.32	0.20***	6.73
Sudden ecological or geological events	−0.14***	−2.93	−0.04**	−2.53	0.09***	2.89	0.08***	3.03
Invasive/alien species or hyper-abundant species	−0.15***	−4.33	−0.04***	−3.13	0.11***	4.74	0.09***	3.96
Köppen Climate Classification A (ref. ‘C’)	−0.05	−0.84	−0.01	−0.80	0.04	0.82	0.03	0.85
Köppen Climate Classification B	0.07	1.19	0.02	1.12	−0.05	−1.18	−0.04	−1.19
Köppen Climate Classification D	−0.11	−1.38	−0.03	−1.43	0.07	1.38	0.06	1.43
Köppen Climate Classification E	−0.09	−0.70	−0.02	−0.65	0.06	0.68	0.05	0.70
Criterion N7	0.11**	2.30	0.03**	2.31	−0.08**	−2.46	−0.07**	−2.27
Criterion N8	0.05	1.19	0.01	1.16	−0.03	−1.20	−0.03	−1.18
Criterion N9	−0.04	−0.89	−0.01	−0.88	0.03	0.89	0.03	0.88
Criterion N10	−0.02	−0.37	0.00	−0.35	0.01	0.37	0.01	0.37
Australia, AU (ref. all other countries)	−0.30	−1.56	−0.08	−1.41	0.21	1.53	0.18	1.57
Bulgaria, BG	−0.23	−1.15	−0.06	−1.14	0.16	1.14	0.13	1.17
Canada, CA	−0.20	−0.97	−0.05	−0.91	0.14	0.96	0.12	0.96
Switzerland, CH	−0.38*	−1.77	−0.10	−1.61	0.26*	1.75	0.22*	1.77
Denmark, DK	−0.66***	−2.91	−0.17**	−2.45	0.45***	2.88	0.38***	2.95
Spain, ES	−0.08	−0.33	−0.02	−0.32	0.05	0.32	0.05	0.32
France, FR	−0.24	−1.22	−0.06	−1.22	0.16	1.22	0.14	1.24
United Kingdom, UK	−0.09	−0.45	−0.02	−0.45	0.06	0.45	0.05	0.45
Greece, GR	0.15	0.55	0.04	0.54	−0.10	−0.55	−0.08	−0.55
Croatia, HR	−0.04	−0.18	−0.01	−0.18	0.03	0.18	0.02	0.19
Italy, IT	−0.53***	−2.86	−0.14**	−2.12	0.36***	2.71	0.30***	2.80
Japan, JP	0.08	0.39	0.02	0.39	−0.05	−0.39	−0.05	−0.39
Norway, NO	−0.19	−1.03	−0.05	−0.98	0.13	1.03	0.11	1.03
New Zealand, NZ	−0.16	−0.75	−0.04	−0.75	0.11	0.75	0.09	0.76
Portugal, PT	−0.14	−0.70	−0.04	−0.70	0.09	0.69	0.08	0.70
Romania, RO	−0.25	−1.11	−0.06	−1.12	0.17	1.11	0.14	1.12
Russia, RU	−0.19	−0.88	−0.05	−0.82	0.13	0.86	0.11	0.87
Slovenia, SI	0.01	0.07	0.00	0.07	−0.01	−0.07	−0.01	−0.07
Turkey, TR	−0.30	−1.51	−0.08	−1.43	0.20	1.49	0.17	1.53
USA, US	−0.44**	−2.27	−0.11*	−1.89	0.30**	2.16	0.25**	2.32
Transboundary	−0.29	−1.49	−0.07	−1.32	0.20	1.47	0.17	1.46
Number of observations	1145							
Number of WHSs (natural and mixed)	83							

The marginal effect dy/dx is calculated for four categories. An Ordered Probit model is estimated using the Stata command ‘oprobit’ and the model option ‘clust(id)’ where id is the world heritage sites. Coefficients and z-stat are displayed in Table 8 in Appendix

***, ** and * indicate statistical significance at the 1, 5 and 10% levels

Even if marginal effects are calculated for all three levels of threats, negligible, minor and major, focus is put on the latter. In this group of threats, the category ‘Climate change and severe weather events’ receives the largest marginal effect of 0.32 (*p* value < 0.01). This implies a 32 percentage points higher probability to perceive this category of threat than the reference group pollution. The likelihood of falling into the group of the most intensive threats is also significantly higher for Invasive/alien species or hyper-abundant species and Local conditions affecting physical fabric (temperatures, rain and dust). Marginal effects of threats related to Social/cultural uses of heritage (tourism/visitor/recreation), Illegal activities and Sudden ecological or geological events are also significantly higher than the reference category. Thus, the marginal effects reflect the descriptive statistics showing that climate change poses the most severe threat to Natural World Heritage Sites, while buildings and development create less pressure. Estimation results also demonstrate that the intensity of threats differs significantly across location, where the highest marginal effects can be observed for Turkey, Italy and Norway (dy/dx = 0.43, 0.38 and 0.34, all with *p* values < 0.01).

Inclusion in the danger list before the year 2014 is positively associated with the extent of threats (*p* value < 0.01). This indicates a certain degree of persistence in the threats to Natural World Heritage Sites. Climate zone is also part of the jigsaw that contributes to the level of threats. Sites in the tropical zone (A) have a higher probability of falling into the high threat category, while sites in the continental/mid-latitude dry climate (D) have the lowest. This is consistent with the expectation that sites in the tropical zone are threatened by multiple factors.

Site characteristics such as size and year of inscription do not play a role in determining the extent of threats to the Natural World Heritage Sites as reported by the management. This is confirmed by a Wald test for joint significance of the age category dummy variables (*p* value of 0.40). The marginal effects for type of site (mixed versus natural) for the two categories negligible and major threat are also not significant at conventional significance levels. In addition, the likelihood of a major threat is significantly lower for criteria N9 (outstanding examples representing significant on-going ecological and biological processes) (dy/dx = −0.07 and *p* value < 0.05) and N7 (dy/dx = −0.08 and *p* value < 0.05).

Overall, hypotheses 1, 3 and 4 relating to location of the site, category and kind of threat cannot be rejected at conventional significance levels, while hypothesis 2 can be partially rejected since only a couple of the criteria are significant (at the 5% level).

These results contradict those of Wang et al. (2015), who find that the threat intensity is highest for pollution, followed by transport infrastructure and physical resource extraction and lowest for climate change and severe weather events, sudden ecological or geological events, and local conditions affecting the physical fabric. It is difficult to identify the reasons for such apparent differences, but one possible underlying explanation could be a growing awareness of the environmental hazard, something that was possibly less pronounced in the dataset from 2004.

In the estimation of the adaptive capacity of the management, marginal effects are calculated for four categories ranging from ‘no capacity’ (lowest category) to ‘high capacity’ (highest category). Results indicate high marginal effects for not being able to deal with climate change threats at 0.20, significant at the 1% level. This implies that the perception of no adaptive capacity for this category is 20 percentage points higher than for the reference category ‘pollution’ (Table 6, for marginal effects and Table 8 in Appendix, for coefficients). Thus, hypothesis 5 stating that external to the management of the site is most difficult to deal with cannot be rejected. Contrary to the estimation of the number of threats, these results do not verify the descriptive statistics, although they are in line with findings by Lemieux et al. (2011) for the Canadian context.

The likelihood of high management adaptive capacity is also lowest for climate change (dy/dx = −0.32, *p* value < 0.01), followed by local conditions affecting physical structure (temperature, rain, dust) (dy/dx = −0.23, *p* value < 0.01) and service infrastructure (dy/dx = −0.10, *p* value < 0.01). A similar order applies to the medium capacity category with marginal effects of −0.11, −0.08 and −0.04, respectively.

The probability of being well-prepared for Sudden ecological or geological events is also lower than that of the reference group. A high adaptive capacity is more common for the categories of buildings and development, transport infrastructure, use/modification of biological resources, extraction of physical resources, all of which are not significantly different from the reference group pollution. Consequently, the results are consistent with earlier literature in that the local management of protected areas deals best with human-induced factors.

High levels of perceived management adaptability are more common for larger sites (*p* value < 0.05) and those that have earlier been on the endangered list (dy/dx = 0.13 and *p* value < 0.05). Mixed sites are significantly less likely to have high management capacity. One explanation behind this could be that sites on the endangered list are implicitly forced to have a higher level of awareness and take action to avoid the risk of losing their UNESCO title. An example outside the economically advanced world is the Galápagos Islands in Ecuador, the first-ever World Heritage Site. Three years after being listed as in danger, appropriate measures were taken by the administration, including a cap on the number of visitors (Lu et al. 2013). Management adaptive capacity also significantly varies across countries with the lowest degree of capacity in the USA, Denmark and Italy.

To test the robustness of the results, a multi-level Ordered Probit model is estimated where the error term is allowed to vary across different threats for a given National Heritage Site. This is an alternative to using cluster-adjusted errors. Results show that the coefficients are quite similar and are thus not reported here. Finally, simpler models are estimated in which the underlying dependent variable is a dummy variable that takes on the value of 1 when a significant threat is present and 0 otherwise. This is estimated using Probit and Logit models. No change in results is achieved by this.

## Conclusions

This study offers new insights into the largest threats to Natural World Heritage Sites in economically advanced countries as considered by their management, based on a large internationally harmonised dataset. It also examines the adaptive capacity of the management to deal with separate threats. Results indicate that the probability to report a major threat to the site is highest for climate change and severe weather events. These threats are also the ones that the management has the lowest capacity to deal with when other important aspects are controlled for.

Data for the analysis mainly consist of official information from UNESCO on 83 Natural and Mixed World Heritage Sites in Europe and the OECD countries, Canada, the United States, Australia, New Zealand, Japan, and South Korea, as well as on 1145 threats sorted in twelve categories.^1^

Estimations of an Ordered Probit model show that besides the high probability of reporting a threat relating to the category of climate change and severe weather events, invasive/alien species or hyper-abundant species and local conditions affecting the physical structure (temperature, rain, dust) are also threat likely perceived. The span between the likelihood of reporting a major climate change threat and a threat from construction and development is 32 percentage points. Factors such as buildings and development, transport infrastructure, service infrastructure, use/alteration of biological resources, physical resource extraction and pollution are least likely to be perceived as a major threat. There are, however, differences across countries, with a higher likelihood of a major threat in Turkey, North America, Norway, and Italy while characteristics of the sites (size, year of inscription, mixed or solely natural site) are all aspects of no significance. Another novel finding is the importance of climate zone for the level of threat. The risk of reporting a major threat is highest for sites in tropical climate zones.

Just like in the case of reporting major threats, climate change is also the category that the management finds themselves having the lowest ability to deal with according to the second set of Ordered Probit estimations. Compared to the reference group (pollution), there is a 20 percentage points higher probability of lacking capacity to deal with such threats. The second highest likelihood of being unable to cope with threats is observed for local conditions affecting the physical structure (temperature, rain, dust) and to a lesser extent for three factors service infrastructure (renewable energy facilities, utilities), sudden ecological or geological events and Invasive/alien species or hyper-abundant species. Site characteristics and year of subscription are also not relevant in most cases. However, the management capacity is higher for sites formerly listed as in anger and lower for mixed sites.

The findings of this study have a number of implications: one is that the kind of threat is more important than site features. This means that most Natural World Heritage Sites are in a similar situation where they are most vulnerable to external threats from climate change at the same time as this is the aspect the management has the lowest capacity to deal with. However, even though mitigating climate change is a global task, this should not prevent site operators from making adaptations to climate change locally as well and from including this task in their long-term planning. The majority of managers report that they have a large or medium level capacity to adapt to human-made threats, such as transportation infrastructure, buildings and development, and the socio-cultural use of heritage (tourism/visitors/recreation), in contrast to climate change. Since there are existing solutions, such as regulating tourist flows and a moratorium on building and infrastructure expansion, these should be prioritised first.

There are not many options available when it comes to climate change and extreme weather, at least not immediately. In the medium and long run promoting eco-friendly modes of transportation to the Natural World Heritage Site and operating the site carbon-neutrally are potential solutions. Visitor promotion of natural World Heritage Sites should be critically considered and reconsidered. Unexpected ecological or geological events are challenging to forecast. Plans for emergencies could be created and improved.

Methodologically, the study highlights the danger of drawing conclusions about relationships based on descriptive statistics. In this case, the estimation results of the most severe threats coincide with the descriptive statistics, but evidence for the management capacity unfolds a slightly different picture when other important aspects are kept constant. Several limitations of this study need to be taken into account. First, the data are cross-sectional, relates to a specific time period and is limited to sites in economically advanced countries. It is likely that threat perceptions change over time. Future work should include the next wave of the period report. Another restriction is that the results are only valid for Natural World Heritage Sites that do indeed report perceived threats. For ten out of 93 sites, information on negative factors is missing and the reason for not reporting is unclear. Given the small proportion of sites not reporting, a possible bias arising from this is expected to be small. Perceptions of threats from a managerial perspective may be subjective and suffer from social desirability bias.

Future work should compare perceptions with satellite data using GIS methods, for instance. Another idea for future research is to extend the dataset to locations in other parts of the world (Asia, Africa, and Latin America). A further approach for new research is to model the determinants of threats based on the Conservation Outlook Assessment which is conducted every third year and covers more than 200 natural sites. However, while there is information on the type of threat by categories, information on the adaptive capacity of the management is not available at the fine-granular level used in this study. Consideration of additional factors, such as topographic features and characteristics of the stakeholders, is also an option.

## Appendix

Tables 7 and 8

**Table 7:** Ordered Probit estimations of the perception of threats to Natural World Heritage Sites

	Coeff.	z-stat
Log size in hectares	0.022	0.68
0–15 years (16–26 years reference category)	−0.118	−0.49
27–32 years	0.013	0.07
33–36 years	0.026	0.15
Danger list	0.658***	2.97
Mixed WHS	−0.200	−1.57
Buildings and development (reference pollution)	−0.027	−0.16
Transportation infrastructure	0.168	0.93
Services infrastructure	0.016	0.10
Biological resource use/modification	0.136	0.77
Physical resource extraction	0.098	0.52
Local conditions affecting physical fabric (temperature, rain, dust)	0.787***	3.68
Social/cultural uses of heritage (tourism/visitor/recreation)	0.578***	2.81
Illegal activities	0.358**	2.00
Climate change and severe weather events	1.210***	6.74
Sudden ecological or geological events	0.755***	3.24
Invasive/alien species or hyper-abundant species	0.851***	4.05
Köppen Climate Classification A (ref. cat. ‘C’)	0.591***	3.54
Köppen Climate Classification B	0.170	1.11
Köppen Climate Classification D	−0.539**	−2.18
Köppen Climate Classification E	−0.130	−0.39
Criterion N7	−0.265**	−2.38
Criterion N8	−0.241	−1.60
Criterion N9	−0.313**	−2.10
Criterion N10	0.104	0.71
Australia, AU (reference all other countries)	0.273	0.73
Bulgaria, BG	−0.881**	−2.42
Canada, CA	1.093***	3.08
Switzerland, CH	1.063**	2.18
Denmark, DK	0.937**	2.03
Spain, ES	0.611	1.63
France, FR	−0.340	−0.92
United Kingdom, UK	0.297	0.85
Greece, GR	0.020	0.04
Croatia, HR	1.068**	2.22
Italy, IT	1.425***	3.82
Japan, JP	0.698*	1.84
Norway, NO	1.309***	3.54
New Zealand, NZ	−0.305	−0.67
Portugal, PT	0.182	0.53
Romania, RO	0.096	0.25
Russia, RU	0.185	0.37
Slovenia, SI	0.742**	2.14
Turkey, TR	1.848***	3.62
USA, US	0.767**	2.18
Transboundary	0.007	0.02
Number of observations (no. of WHSs (natural and mixed))	1145 (83)	
Pseudo *R*^2^	0.175	

dy/dx denotes average marginal effects. The ordered Probit model is estimated using the Stata command ‘oprobit’ and the model option ‘clust(id)’ where id is the world heritage sites. The cutoff points 1 and 2 are −0.234 and 1.326, respectively, the latter being significant at the 1% level

***, ** and * indicate statistical significance at the 1, 5 and 10% significance level, respectively

**Table 8:** Ordered Probit estimations of the management capacity to deal with threats to World Heritage Sites

	Coeff.	z-stat
Log size in hectares	0.134**	1.95
0–15 years (16–26 years reference category)	0.091	0.25
27–32 years	0.126	0.38
33–36 years	−0.003	−0.01
Danger list	0.550**	2.14
Mixed WHS	−0.637***	−3.11
Buildings and development (reference pollution)	0.012	0.07
Transportation infrastructure	−0.164	−1.23
Services infrastructure	−0.609***	−4.26
Biological resource use/modification	−0.143	−0.88
Physical resource extraction	−0.263	−1.26
Local conditions affecting physical fabric (temperature, rain, dust)	−0.999***	−4.84
Social/cultural uses of heritage (tourism/visitor/recreation)	−0.343**	−2.00
Illegal activities	−0.373*	−1.92
Climate change and severe weather events	−1.489***	−8.02
Sudden ecological or geological events	−0.594***	−3.02
Invasive/alien species or hyper-abundant species	−0.668***	−4.72
Köppen Climate Classification A (ref. cat. ‘C’)	−0.223	−0.84
Köppen Climate Classification B	0.309	1.18
Köppen Climate Classification D	−0.472	−1.39
Köppen Climate Classification E	−0.397	−0.69
Criterion N7	0.491**	2.37
Criterion N8	0.214	1.19
Criterion N9	−0.191	−0.89
Criterion N10	−0.082	−0.37
Australia, AU (reference all other countries)	−1.320	−1.55
Bulgaria, BG	−0.993	−1.16
Canada, CA	−0.879	−0.96
Switzerland, CH	−1.638*	−1.77
Denmark, DK	−2.855***	−2.96
Spain, ES	−0.343	−0.33
France, FR	−1.027	−1.23
United Kingdom, UK	−0.379	−0.45
Greece, GR	0.636	0.55
Croatia, HR	−0.170	−0.18
Italy, IT	−2.292***	−2.84
Japan, JP	0.345	0.39
Norway, NO	−0.822	−1.03
New Zealand, NZ	−0.697	−0.75
Portugal, PT	−0.590	−0.70
Romania, RO	−1.075	−1.11
Russia, RU	−0.824	−0.87
Slovenia, SI	0.054	0.07
Turkey, TR	−1.293	−1.51
USA, US	−1.885**	−2.25
Transboundary	−1.257	−1.49
Number of observations (number of WHS)	1145 (83)	
Pseudo *R*^2^	0.160	

dy/dx denotes average marginal effects. See Table 7

***, ** and * indicate statistical significance at the 1, 5 and 10% significance levels

## Footnotes

1 There is no natural or mixed world heritage site in Israel.
